# The Apolipoprotein B/Apolipoprotein A-I Ratio as a Potential Marker of Plasma Atherogenicity

**DOI:** 10.1155/2015/591454

**Published:** 2015-03-23

**Authors:** Anastasiya M. Kaneva, Natalya N. Potolitsyna, Evgeny R. Bojko, Jon Ø. Odland

**Affiliations:** ^1^Institute of Physiology, Komi Science Center, Ural Branch of Russian Academy of Sciences, Pervomaiskaya Avenue 50, Syktyvkar 167982, Russia; ^2^Faculty of Health Sciences, The Arctic University of Norway, 9037 Tromsø, Norway

## Abstract

*Background.* The apolipoprotein (apo) B/apoA-I ratio represents the balance between apoB-rich atherogenic particles and apoA-I-rich antiatherogenic particles, and this ratio is considered to be a marker of cardiovascular risk. Although many studies have demonstrated the importance of the apoB/apoA-I ratio in predicting the presence or absence of cardiovascular disease, less is known about apoB/apoA-I ratio as a marker of plasma atherogenicity. *Methods.* A total of 157 normolipidemic men aged 20–59 years were included in the study. The plasma levels of total cholesterol (TC), triglycerides (TG), high-density lipoprotein cholesterol (HDL-C), apoA-I, apoB, and apoE were determined after a 12 h fasting period. *Results*. The median of the apoB/apoA-I ratio in the studied normolipidemic subjects was 0.52, with values ranging from 0.19 to 2.60. The percentage of subjects with the apoB/apoA-I ratio exceeding 0.9 (the accepted risk value of cardiovascular disease) was 19.1%. The subjects with apoB/apoA-I>0.9 were characterized by higher TG levels and atherogenic index of plasma (AIP) and lower values of ratio of low-density lipoprotein cholesterol (LDL-C) to apoB (LDL-C/apoB) and apoE levels compared with men with apoB/apoA-I<0.9. *Conclusion*. Despite normolipidemia, the subjects with the unfavorable apoB/apoA-I ratio had more atherogenic lipid profile.

## 1. Introduction

Atherosclerosis is a pathologic process affecting blood vessels, which leads to the development of cardiovascular disease [1, 2]. Although atherogenesis is a multifactorial process, abnormalities in lipid metabolism are a key factor, representing approximately 50% of the population-attributable risk of developing cardiovascular disease [3]. The recent studies have shown that using the conventional lipid indices can result in errors in the assessment of cardiovascular risk [4–6]. A considerable proportion of patients with atherosclerotic disease has levels of total cholesterol (TC) and low-density lipoprotein cholesterol (LDL-C) within the health-related reference interval [7, 8], and some patients who achieve significant LDL-C reduction with lipid-lowering therapy still develop cardiovascular disease [9, 10].

The main cause of errors in the estimation of the lipoprotein-related risk of cardiovascular disease when using the conventional lipid indexes is due to the wide variance of cholesterol within the low-density lipoprotein and high-density lipoprotein molecules; this variance is due to the active exchange of lipid components between lipoproteins. Hence, the lipoprotein cholesterol concentration does not always correspond to lipoprotein concentration [11, 12]. Unlike lipoprotein cholesterol, the lipid-transporting apolipoprotein (apo), apoB, remains with the lipoproteins without undergoing any changes [12]. ApoB is an essential structural component of very low-density lipoproteins, intermediate-density lipoproteins, and low-density lipoproteins. Because each particle of these lipoproteins contains one molecule of apoB, the total atherogenic particles number can be accurately estimated by measuring the plasma level of this apoprotein. In contrast, apoA-I, the major structural constituent of antiatherogenic high-density lipoproteins, exchanges between lipoproteins and the number of apoprotein molecules varies between lipoprotein particles. Nevertheless, levels of apoA-I in plasma are strongly correlated with HDL levels. Therefore, apoB and apoA-I are assumed to be superior markers for lipoprotein abnormalities [11, 13–16]. The blood levels of apoA-I and apoB in patients with cardiovascular disease have been shown to be better discriminators than high-density lipoprotein cholesterol (HDL-C) and LDL-C levels [8, 17]. For example, in cases where LDL-C is in the health-related reference interval, high apoB levels may indicate an increased number of small, dense low-density lipoprotein particles, which are the most atherogenic particles [18–20].

Although the concentrations of apoB and apoA-I are associated with cardiovascular disease more strongly than the corresponding lipoprotein cholesterol fractions, the discriminant value of these apoproteins in absolute terms appears to be less than that of their ratio (the apoB/apoA-I ratio) [11]. The apoB/apoA-I ratio reflects the balance of atherogenic and antiatherogenic lipoproteins in plasma [4, 14–16]. Multiple clinical and epidemiological studies have confirmed that the apoB/apoA-I ratio is a superior marker for cardiovascular disease compared with lipids and lipoproteins or their ratios [2–4, 23–29]. Therefore, some studies demonstrated that the apoB/apoA-I ratio could discriminate between patients with coronary artery disease (CAD) and those without, even when the CAD patients had normal lipid levels [30]. The cut-off values for the apoB/apoA-I ratio that define a high cardiovascular risk were proposed to be 0.9 for men and 0.8 for women [14, 29].

In this study, we determined the variation limits of the apoB/apoA-I ratio in healthy middle-aged men with normolipidemia and the relationship of this ratio with other lipid indexes.

## 2. Materials and Methods

### 2.1. Subjects and Sampling

Our study included 157 apparently healthy men with normolipidemia. Anthropometric measurements (height and weight) and other personal information were obtained during the clinical examination and the interview. Weight was measured to the nearest 100 g using a standard medical scale while the subjects were minimally clothed without shoes. Height was measured to the nearest 0.5 cm in a standing position without shoes using a standard stadiometer. The body mass index was calculated as the weight (in kilograms) divided by the square of the height (in meters).

Participants were excluded from the study using the following criteria: (i) a body mass index of 30 kg/m^2^ or greater; (ii) a TC concentration above 5.2 mmol/L; (iii) a triglycerides (TG) concentration above 1.8 mmol/L; and (iv) a HDL-C concentration below 0.9 mmol/L. All participants were considered to be free from serious and chronic illnesses at the time of the recruitment. Each subject gave written informed consent for participating in the study, which was approved by the Ethics Committee of Institute of Physiology, Komi Science Center, Ural Branch of Russian Academy of Sciences.

Fasting blood samples were taken from the antecubital vein into vacutainers (Becton Dickinson BP). Blood samples were centrifuged and plasma was placed into Eppendorf microcentrifuge tubes and was stored at −40°C until analysis.

### 2.2. Lipid Measurements

The plasma TC and TG concentrations were measured using an enzymatic method with commercially available kits (Chronolab Systems, S.L. Barcelona, Spain). The HDL-C concentration was determined by assaying the cholesterol in the supernatant obtained after the precipitation of apoB-containing lipoproteins with phosphotungstate/magnesium chloride. The plasma apoA-I, apoB, and apoE concentrations were measured using an immunoturbidimetric method with kits (Chronolab Systems, S.L. Barcelona, Spain). The samples were analyzed immediately after thawing at 37°C in a thermostatic bath. The measurement of each sample was carried out in duplicate, and the mean was calculated. The absorbance of all samples was measured on the PowerWave 200 automated spectrophotometer (BioTek Instruments, USA).

The LDL-C was calculated according to Friedewald's formula [31]. The non-HDL-C was calculated as the difference between the TC and HDL-C. Some clinical indicators of lipids metabolism were computed, including the TC/HDL-C, apoB/apoA-I, and LDL-C/apoB ratios, as well as atherogenic index of plasma (AIP).

For calculating the LDL-C/apoB ratio, the concentration of LDL-C is transformed of mmol/L in mg/dL. AIP was calculated as a logarithm of the ratio of the molar concentration (mmol/L) of TG to HDL-C (i.e., log [TG/HDL-C]) [32].

### 2.3. Statistical Analysis

The statistical analysis was performed using Statistica 6.0 (Statsoft, Tulsa, USA). The continuous variables in the tables are presented as the median and interquartile range (25th and 75th percentiles). Differences between the groups were analyzed using the Mann-Whitney test and the chi-squared test. Correlations between the indices were assessed using the Spearman's rank correlation. A value of *P* < 0.05 was accepted as statistically significant.

## 3. Results

The main characteristics of the studied subjects are shown in Table 1. The subjects ranged in age from 20 to 59 years with the average age of 36.0 years. The median for body mass index in the studied men was 24.8 kg/m^2^.

**Figure 1:**
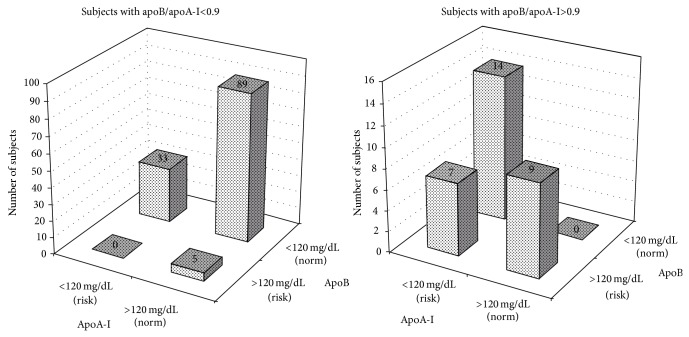
Distribution of subjects depending on the apoA-I and apoB levels in the groups with apoB/apoA-I<0.9 and apoB/apoA-I>0.9. Values of apoB>120 mg/dL and apoA-I<120 mg/dL have been proposed as the cut-off points defining a high cardiovascular risk [21, 22].

The median value (25%; 75%) of the apoB/apoA-I ratio for the entire group was 0.52 (0.42; 0.78), with values ranging from 0.19 to 2.60. By virtue of the exclusion criteria, the plasma TC, TG, and HDL-C levels in the men varied in the health-related reference interval. Nevertheless, of the 157 studied subjects, 30 men (19.1%) had the apoB/apoA-I ratio above 0.9. The values of apoB and apoA-I varied from 30.0 to 218.0 mg/dL and from 50.0 to 300.0 mg/dL, respectively. According to the health-related reference values of apoproteins [21, 22], low values of apoA-I were determined in 14 subjects, and high values of apoB were found in 9 men of 30 subjects with the unfavourable apoB/apoA-I ratio. Seven subjects had low levels of apoA-I along with high levels of apoB. In the group of men with apoB/apoA-I<0.9, low values of apoA-I and high values of apoB were observed in 33 and 5 subjects (26.0% and 3.1% of the total cases), respectively (Figure 1).

Table 2 compares the subjects with apoB/apoA-I<0.9 with those with apoB/apoA-I>0.9. There were expected differences (due to the way in which the study was designed) between the groups in their apoB and apoA-I levels and the ratios in which the apoproteins were used as components. The group of the subjects with apoB/apoA-I>0.9 was characterized by higher TG levels (+27.6%; *P* = 0.004) and AIP (+50.0%; *P* = 0.032) and lower apoE levels (−22.1%; *P* = 0.011) compared to the group of the men with apoB/apoA-I<0.9 (Table 2). The TC, HDL-C, LDL-C, non-HDL-C levels, and TC/HDL-C ratio did not significantly differ between the two groups. Nevertheless, of note, the subjects with the high apoB/apoA-I ratio were more likely to have an atherogenic lipid profile. Reduced levels of apoA-I in the men with the unfavourable apoB/apoA-I ratio against the background of invariable concentration of HDL-C may indicate a decline of the functional properties of high-density lipoproteins. A significant decrease of the LDL-C/apoB ratio in the subjects with apoB/apoA-I>0.9 (compared with those with apoB/apoA-I<0.9) reflected the presence of small, dense low-density lipoprotein particles.

Using Spearman's rank correlation (Table 3), TG, apoE, and AIP were found to be significantly correlated with the apoB/apoA-I ratio. No correlation was observed between the apoB/apoA-I ratio and the levels of TC, HDL-C, LDL-C, and non-HDL-C or TC/HDL-C ratio. ApoA-I did not reveal a significant positive or negative correlation with any lipid parameters. As expected, the plasma levels of apoB were positively associated with the TC, TG, and non-HDL-C levels, as well as the TC/HDL-C ratio and AIP. A significant negative correlation was also observed between apoB and apoE.

**Figure 2:**
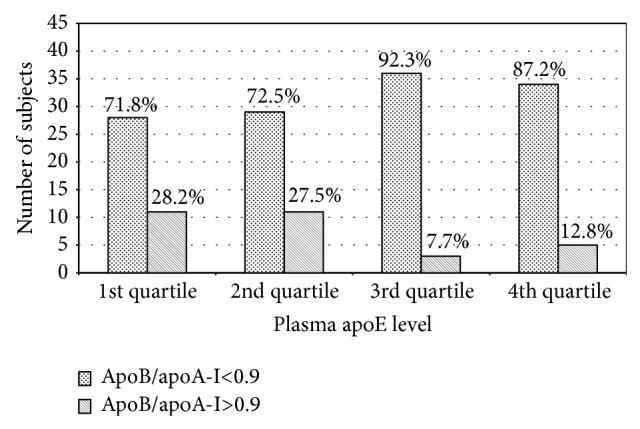
Stratification of the subjects according to the quartiles of apoE levels and the cut-off values of the apoB/apoA-I ratio. 1st quartile: apoE<1.91 mg/dL; 2nd quartile: apoE from 1.91 to 2.61 mg/dL; 3rd quartile: apoE from 2.62 to 3.46 mg/dL; 4th quartile: apoE > 3.46 mg/dL.

The relationship between the values of the apoB/apoA-I ratio and the plasma apoE levels was evaluated using the stratification of the subjects according to the quartiles of apoE levels, as well as the apoB/apoA-I ratio values that have been proposed to define increased cardiovascular risk (cut-off values >0.9) (Figure 2). The unfavourable apoB/apoA-I ratio was more frequently observed in the subjects with the low levels of apoE (1st and 2nd quartiles). The percentage of the subjects with apoB/apoA-I>0.9 decreased significantly with the progressive increase in apoE levels (*χ* ^2^ = 8.20; *P* = 0.042). In particular, in the 1st and 2nd quartiles, these percentages were 28.2% and 27.5%, respectively; in the 3rd and 4th quartiles, they decreased to 7.7% and 12.8%, respectively.

## 4. Discussion

The atherogenic lipid profile is an important risk factor for cardiovascular disease. This profile is characterized by elevated levels of TC, LDL-C, and TG and lowered levels of HDL-C [33, 34]. However, these traditional lipid risk factors are not always adequate indicators of cardiovascular risk [35]. The most recent studies have shown that the diagnostic accuracy of the apoB/apoA-I ratio is significantly greater than that of any lipid parameters [2–4, 23–29].

A total of 19.1% of the studied subjects with normolipidemia had the apoB/apoA-I ratio that exceeded 0.9. The epidemiological studies have revealed that a higher apoB/apoA-I ratio indicates a higher cardiovascular risk, such that the cut-off value of 0.9 has been proposed to define a risk of developing cardiovascular disease [14]. Thus, our results have shown that the unfavourable values of the apoB/apoA-I ratio may be observed in subjects without atherogenic changes in their lipid profile. Similar data have been reported by other authors [36–39]. The results of these clinical cross-sectional and prospective studies have shown that the apoB/apoA-I ratio was the only lipid-related variable that differentiated normolipidemic patients with CAD from those without CAD.

The lipoprotein-related risk is associated with the overall balance between the atherogenic and antiatherogenic lipoproteins. Several lipoprotein and apoprotein ratios or “atherogenic indices” have been defined to express this balance. The TC/HDL-C, apoB/apoA-I, and AIP are the most well-known ratios indicating the balance between the atherogenic and protective lipoproteins [35]. In the studied subjects, the apoB/apoA-I ratio was correlated with AIP. However, no relationship was found between the apoB/apoA-I and TC/HDL-C ratios. It is believed that the TC/HDL-C ratio indicates the proportion of atherogenic to antiatherogenic lipoproteins. However, the cholesterol levels in lipoproteins do not always accurately reflect the concentrations of the plasma lipoproteins. This may occur during changes in the size and composition of the lipoprotein particles [4]. The absence of correlation between the apoB/apoA-I and TC/HDL-C ratios in the studied subjects indicates a difference in the predictive power of these ratios. At the same time, the significant positive correlation between the apoB/apoA-I ratio and AIP was found. AIP, calculated as log [TG/HDL-C], reflects the balance between atherogenic and protective lipoproteins and theoretically depicts the high-density lipoproteins esterification rate and low-density lipoproteins size. High AIP indicates an increase in the percentage of small high-density lipoproteins and small, dense low-density lipoprotein particles in plasma [32]. Thus, AIP reflects the qualitative composition of lipoproteins, while the apoB/apoA-I ratio shows their number. The correlation between the apoB/apoA-I ratio and AIP indicates that simultaneous changes in the number and composition of lipoproteins were observed in the studied subjects.

Our data have demonstrated that an increase in apoB values in the subjects with apoB/apoA-I>0.9 was not accompanied by a rise in the LDL-C levels. As a result, the LDL-C/apoB ratio in the subjects with apoB/apoA-I>0.9 was twofold less than in those with apoB/apoA-I<0.9. The low values of the LDL-C/apoB ratio indicate the predominance of small, dense low-density lipoprotein particles in plasma [40]. ApoB is well known to be the major apoprotein in all potentially atherogenic lipoprotein particles. However, more than 90% of all apoB in the blood is found in low-density lipoproteins. Therefore, the LDL-C/apoB ratio provides approximate information on low-density lipoprotein particles size [14, 35, 41]. Small, dense low-density lipoproteins are considered to promote atherosclerosis because of their low affinity for low-density lipoprotein receptors and susceptibility to oxidative modification [42]. Thus, our results indicate that the high apoB/apoA-I ratio in the normolipidemic subjects was associated with an increase in lipid profile atherogenicity, which occurred due to changes in the low-density lipoprotein particles size and composition.

The present study has showed that the subjects with apoB/apoA-I>0.9 were also characterized by higher levels of TG and lower concentrations of apoE. This finding indicates a disturbance in the clearance of TG-rich lipoproteins. ApoE is known to mediate the uptake of TG-rich lipoprotein particles via receptor-related endocytosis. There is an optimal concentration of the plasma apoE that is required for lipoprotein clearance, and too little or too much of the protein can be detrimental. Too little apoE impairs the clearance of TG-rich lipoproteins and their remnants from plasma. Too much apoE stimulates very low-density lipoproteins production in the liver and impairs lipoprotein lipase-mediated lipolysis, leading to hypertriglyceridemia [43, 44]. We have previously shown that the low apoE levels may contribute to hypertriglyceridemia in residents of the European North of Russia [45]. The results of the present study have indicated a relationship between the unfavourable apoB/apoA-I ratio and the low plasma apoE levels (Figure 2). Moreover, the significant negative correlation between apoE and apoB was found (Table 3). Thus, an increase in the apoB/apoA-I ratio in the studied subjects may be assumed to be accompanied by the accumulation of TG-rich lipoproteins in plasma, which was associated with the reduced plasma very low-density lipoproteins clearance due to the low levels of apoE.

## 5. Conclusions

The apoB/apoA-I ratio exceeding 0.9 may be observed even in the subjects with normolipidemia. An increase in the apoB/apoA-I ratio was accompanied by a rise of AIP, which indicated simultaneous changes in the number and composition of lipoproteins in the studied subjects. The subjects with apoB/apoA-I>0.9 were also characterized by higher levels of TG and lower values of LDL-C/apoB ratio and concentrations of apoE compared with the subjects with apoB/apoA-I<0.9. On the whole, the subjects with apoB/apoA-I>0.9 had more atherogenic lipid profile. Thus, the apoB/apoA-I ratio can be considered as a sensitive marker of atherogenic risk.

## Figures and Tables

**Table 1:** Baseline characteristics of study participants (*n* = 157).

	Median (25%; 75%)	Min–Max
Age, years	36.0 (29.0; 45.0)	20–59
Body mass index, kg/m^2^	24.8 (22.6; 26.5)	18.0–29.9
TC, mmol/L	4.13 (3.58; 4.64)	2.73–5.15
TG, mmol/L	1.11 (0.87; 1.38)	0.56–1.70
HDL-C, mmol/L	1.40 (1.21; 1.56)	1.00–1.99
LDL-C, mmol/L	2.21 (1.66; 2.69)	0.42–3.68
Non-HDL-C, mmol/L	2.74 (2.14; 3.26)	1.01–4.11
TC/HDL-C	2.93 (2.48; 3.45)	1.57–4.99
ApoA-I, mg/dL	149.8 (100.8; 180.0)	50.0–300.0
ApoB, mg/dL	78.0 (56.1; 102.0)	30.0–218.0
ApoB/apoA-I	0.52 (0.42; 0.78)	0.19–2.60
ApoE, mg/dL	2.61 (1.91; 3.46)	0.48–5.54
LDL-C/apoB	1.06 (0.73; 1.46)	0.20–2.48
AIP	−0.09 (−0.22; −0.01)	−0.45–0.22

Values are presented as the median, interquartile range, minimum, and maximum.

**Table 2:** The plasma lipids and apolipoproteins in the groups with apoB/apoA-I<0.9 (*n* = 127) and apoB/apoA-I>0.9 (*n* = 30).

	Group with apoB/apoA-I<0.9	Group with apoB/apoA-I>0.9	*P* value^*^
Age, years	35.0 (29.0; 43.0)	39.0 (30.0; 47.0)	0.185
Body mass index, kg/m^2^	24.7 (22.7; 26.5)	24.8 (21.9; 25.7)	0.813
TC, mmol/L	4.12 (3.55; 4.64)	4.24 (3.58; 4.73)	0.825
TG, mmol/L	1.05 (0.85; 1.34)	1.34 (1.06; 1.65)	**0.004**
HDL-C, mmol/L	1.40 (1.21; 1.55)	1.41 (1.25; 1.58)	0.420
LDL-C, mmol/L	2.30 (1.66; 2.70)	2.10 (1.60; 2.64)	0.478
Non-HDL-C, mmol/L	2.74 (2.14; 3.26)	2.71 (2.06; 3.28)	0.943
TC/HDL-C	2.93 (2.50; 3.51)	2.92 (2.36; 3.25)	0.538
ApoA-I, mg/dL	152.6 (115.0; 188.0)	102.6 (73.0; 122.4)	**<0.001**
ApoB, mg/dL	71.0 (53.0; 89.0)	147.9 (98.9; 190.0)	**<0.001**
ApoB/apoA-I	0.48 (0.38; 0.62)	1.36 (1.07; 1.61)	**<0.001**
ApoE, mg/dL	2.81 (2.00; 3.55)	2.19 (1.23; 2.96)	**0.011**
LDL-C/apoB	1.16 (0.88; 1.59)	0.61 (0.43; 0.69)	**<0.001**
AIP	−0.10 (−0.23; −0.01)	−0.05 (−0.15; 0.04)	**0.032**

Values are presented as the median and interquartile range. ^*^The Mann-Whitney *U* test was used to estimate differences between the groups.

**Table 3:** Spearman correlation coefficients between the apoA-I, apoB, apoB/apoA-I ratio, and other lipid parameters (*n* = 157).

	ApoA-I		ApoB		ApoB/apoA-I ratio	
	Spearman, *r*	*P*-level	Spearman, *r*	*P*-level	Spearman, *r*	*P*-level
TC	0.06	0.443	**0.17**	**0.031**	0.06	0.421
TG	−0.04	0.577	**0.41**	**<0.001**	**0.32**	**<0.001**
HDL-C	0.07	0.335	−0.06	0.426	−0.07	0.362
LDL-C	0.08	0.301	0.12	0.125	0.04	0.655
Non-HDL-C	0.08	0.312	**0.21**	**0.010**	0.09	0.240
TC/HDL-C	0.08	0.302	**0.21**	**0.009**	0.12	0.139
ApoE	−0.01	0.867	−**0.28**	**<0.001**	−**0.19**	**0.012**
AIP	−0.02	0.797	**0.42**	**<0.001**	**0.33**	**<0.001**

Data are correlation coefficients and *P* values. The Spearman's rank correlation was used.

## Abbreviations

Apo: Apolipoprotein
AIP: Atherogenic index of plasma
CAD: Coronary artery disease
HDL-C: High-density lipoprotein cholesterol
LDL-C: Low-density lipoprotein cholesterol
TC: Total cholesterol
TG: Triglycerides.
